# Association Between Helicobacter pylori Eradication and Kidney Function in Patients With Chronic Gastritis: A Retrospective Single-Center Study

**DOI:** 10.7759/cureus.21621

**Published:** 2022-01-26

**Authors:** Emad Aljahdli, Sahar J Almaghrabi, Talal L Alhejaili, Waleed Alghamdi

**Affiliations:** 1 Internal Medicine, King Abdulaziz University Hospital, Jeddah, SAU; 2 Medical Student, King Abdulaziz University Hospital, Jeddah, SAU; 3 Internal Medicine-Gastroenterology, King Abdulaziz University Hospital, Jeddah, SAU

**Keywords:** chronic gastritis, renal function, serum creatinine, helicobacter pylori, helicobacter pylori eradication, gfr

## Abstract

Background

*Helicobacter pylori* (HP) is a common bacterium that globally infects humans. The significance of HP infection and eradication of kidney impairment remain ambiguous. Moreover, little is known about whether elimination of the bacteria has any consequence on kidney function. This study aimed to explore the relationship between HP eradication and kidney function in patients with chronic gastritis (CG).

Methodology

We retrospectively reviewed the records of all CG patients with eradicated HP at King Abdulaziz University Hospital between June 2002 and June 2021. All patients older than 18 years, diagnosed with CG in whom HP had been eradicated, were included. Out of 1,936 patients’ records, only 46 met the criteria.

Results

The mean age of the study sample was 48 years; in addition, 58.7% of the patients were obese. There was no significant difference in serum creatinine, blood urea nitrogen, and glomerular filtration rate after HP eradication (P-values of 0.414, 0.112, and 0.300, respectively).

Conclusions

We found no relationship between the eradication of HP and improvement in renal function. However, prospective population-based studies must be conducted to assess an association between HP eradication and renal function, as well the future risk of nephropathy with the persistence of HP. As such, we recommend a multicenter study that includes a representative sample size.

## Introduction

*Helicobacter pylori* (HP) is a spiral-shaped, microaerophilic Gram-negative flagellated bacterium that usually lives in the gastric mucosa [1-3]. HP infection is a common bacterial infection that affects humans globally. Approximately 50% of the population is infected with HP, with infection levels surpassing 70% in some developing areas [3-5].

Chronic gastritis (CG) is a long-standing inflammation of the gastric mucosa, combined with different degrees of damage of superficial and glandular epithelia. The causes of CG are exogenous (mainly HP) and endogenous [6].

Blood tests for blood urea nitrogen (BUN) and creatinine are the easiest way to monitor kidney function. These are normal metabolic waste products excreted by the kidneys. BUN is an indirect and rough measurement of renal and liver function measuring the amount of urea nitrogen in the blood. BUN is related to the excretory function of the kidney [7]. Serum creatinine (SC) is widely used as a measure of kidney function; however, the serum level reflects renal excretion, as well as the generation, intake, and metabolism of creatinine [8]. SC is used to calculate glomerular filtration rate (GFR), which is the most accurate way to measure renal function.

However, the importance of HP infection and renal function remains unclear. HP is an etiological factor in endothelial dysfunction and may be involved in causing proteinuria by this mechanism. Little is known about whether eradicating the bacteria would have any impact on renal function [3]. Future population-based studies regarding whether the elimination of HP infection could decrease the risk of end-stage renal disease (ESRD) are warranted [9].

In their study, Pan et al. indicated that in patients with peptic ulcer disease, HP infection is probably a risk factor associated with renal injury. Moreover, HP eradication may decrease the risk of renal damage and prevent chronic kidney disease (CKD) [10].

To our knowledge, there are no studies addressing this issue, considering different population traits with other studied populations. We hypothesize that HP eradication has a positive impact on kidney function, demonstrated by estimated GFR. We identified CG at King Abdulaziz University Hospital (KAUH), which may be a new HP eradication goal if proven.

## Materials and methods

This is a retrospective record review study of all CG patients infected with HP, who underwent treatment and eradication at KAUH between June 2002 and June 2021. Medical records of 1,936 adult patients were reviewed, with a total of 46 records meeting the criteria. Ethical approval was obtained from the Research Ethics Committee Board, KAUH, College of Medicine (reference no. 162-21).

In collaboration with the Pathology Department at KAUH, all record numbers for patients diagnosed between 2002 and 2021 (with HP-induced CG based on histopathology results) were retrieved. Medical records with 1,936 patients were subsequently reviewed. In addition, patient data collected through the official KAUH medical record database (Phoenix) between June and August 2021 for eligibility, based on pre-specified inclusion and exclusion criteria, were assessed. To reiterate, 46 records were recruited for analysis.

*H. pylori* eradication test

We used the urea breath test (UBT), histopathology reports, or HP stool antigens to confirm the diagnosis, as well as the eradication of HP infection.

Population and data collection

All patients 18 years or older, diagnosed with HP-induced CG, followed by treatment and eradication, were included in the study. Patients who were aged <18 years or had a history of malignancy, renal impairment, or missing data were excluded. Data from KAUH included pertinent demographics: age, gender, nationality, height, weight, pre- and post-eradication SC, and BUN during 2021. Reference ranges are listed in Table 1, according to the KAUH laboratory.

**Table 1:** Reference ranges according to the KAUH lab. SC: serum creatinine; BUN: blood urea nitrogen; GFR: glomerular filtration rate; KAUH: King Abdulaziz University Hospital

Laboratory test	Reference range
SC	53-115 µmol/L
BUN	2.5-6.4 mmol/L
GFR	>60 mL/minute

Estimated GFR was calculated using a four-variable MDRD Study Equation [11]: GFR = 175 × (SC)-1.154 × (age)-0.203 × 0.742 [if female] × 1.212 [if Black] is SC in mg/dL.

Statistical analysis

Microsoft Excel 2019 was used for data entry, with statistical analysis performed using SPSS Statistics version 21 (IBM Corp., Armonk, NY, USA). Continuous variables are displayed using means and standard deviations, whereas categorical variables are depicted with frequencies and percentages. Analysis of variance and the paired sample t-test were used in the analysis. P-values of <0.05 were considered statistically significant.

## Results

There were 46 CG patients with eradicated HP. In addition, 56.5% were female, 58.7% were obese, and 63% were Saudi nationals. Table 2 shows the mean and standard deviation (SD) for age and body mass index (BMI), with gender and nationality presented in frequency and percentage.

**Table 2:** Anthropometric and demographic data of study participants. BMI: body mass index; SD: standard deviation

Features		Results
Age (mean ± SD)		48.74 ± 12.2
Gender (n, n%)	Female	26 (56.5%)
	Male	20 (43.5%)
Nationality (n, n%)	Saudi	29 (63%)
	Non-Saudi	17 (37%)
BMI (mean ± SD)		33.87 ± 8.9

Descriptive statistics were calculated for all lab tests that reflect kidney function (Table 3). The mean levels of SC and BUN decreased after eradication, whereas the GFR mean level was higher after eradication.

**Table 3:** Mean and SD of lab tests to measure kidney function. SC: serum creatinine; BUN: blood urea nitrogen; GFR: glomerular filtration rate; SD: standard deviation

Laboratory test	Mean ± SD
Pre-SC	69.20 ± 13.97
Post-SC	67.94 ± 15.54
Pre-BUN	4.26 ± 1.39
Post-BUN	3.90 ± 1.38
Pre-GFR	92.72 ± 20.24
Post-GFR	95.28 ± 26.48

A paired sample t-test was performed for SC, BUN, GFR, and pre- and post-HP eradication, with p-values of 0.414, 0.112, and 0.300, respectively. There was no significant difference in SC, BUN, and GFR after HP eradication (Table 4).

**Table 4:** Paired sample t-test. SC: serum creatinine; BUN: blood urea nitrogen; GFR: glomerular filtration rate

Laboratory test	P-value
Pre- and post-SC	0.414
Pre- and post-BUN	0.112
Pre- and post-GFR	0.300

## Discussion

The present study revealed no association between HP eradication and renal function. Similar results were reported by Alimadadi et al. in a clinical trial on HP eradication regarding creatinine clearance in patients with peptic ulcer disease [12]. Kong et al. conducted a cross-sectional study to evaluate the association between HP infection and kidney disease among Chinese adults [13]. They found that decreased GFR levels were not significantly different between HP-positive and negative subjects. Wijarnpreecha et al. conducted a meta-analysis on the association of HP with CKD [14]. Their study showed no relationship between HP infection and non-dialysis-dependent kidney disease and CKD. Wang et al. conducted a population-based cohort study on the association between HP eradication and CKD in patients with peptic ulcer disease [3]. They compared cohort B, in whom HP was not eradicated, to cohort A, in whom HP was eradicated.

In contrast, other authors found an association between HP infection and renal dysfunction [9,10,15]. Lin et al. conducted a national population-based cohort study on the association between HP infection and the subsequent risk of ESRD [9]. Their results showed that the incidence of ESRD was 3.72 times higher in the HP-infected cohort than in the non-infected cohort. Pan et al. conducted a prospective cohort study on the link between HP infection and kidney damage in patients with peptic ulcers [10]. Their results demonstrated a positive correlation between HP infection and albumin creatinine ratio abnormality. Chiu et al. conducted a population-based cohort study in Taiwan on the risk of CKD after early and late HP eradication in patients with peptic ulcer disease [15]. They found that subsequent CKD was 1.44-fold higher in the late eradication cohort than the early eradication cohort. A meta-analysis done by Shin et al. evaluated the prevalence of HP infection in patients with CKD and concluded a lower prevalence of HP infection in patients with CKD [16]. Some researchers found that gastric and extra-gastric HP infections played a notable role in advancing systemic disease, mainly renal dysfunction and ESRD [17]. In a study by Satoh, HP infection was reported to subsidize endothelial dysfunction, being directly linked to the progression of CKD and declining renal function [18]. The study found no significant difference in CKD occurrence between the two arms. Several studies with different designs have been conducted to draw a clear conclusion; however, results have been conflicting [3,9,10,12-18].

The limitation of our study includes missing data, given a retrospective design that limits available readings of SC and BUN. The small and less representative sample size because the study was done in one health care center. Some strengths of this study include excluding all malignancy and renal disease that might have affected the SC level. Finally, increasing the number of patients in the study, using different modalities to confirm HP eradication (with histopathology reports), and noting HP stool sample antigens with the UBT could have helped.

## Conclusions

We found no association between HP eradication and improvement in renal function. Yet, prospective population-based studies must be conducted to assess an association between HP eradication and renal function, especially if there is a risk of nephropathy with HP persistence. We recommend a multicenter study design that would be a representative sample size.
